# Radiofrequency Catheter Ablation of Supraventricular Tachycardia in Patients With Pulmonary Hypertension: Feasibility and Long-Term Outcome

**DOI:** 10.3389/fphys.2021.674909

**Published:** 2021-06-18

**Authors:** Bin Zhou, Yong-Jian Zhu, Zheng-Qin Zhai, Si-Xian Weng, Ya-Zhe Ma, Feng-Yuan Yu, Ying-Jie Qi, Yi-Zhou Jiang, Xin Gao, Xi-Qi Xu, Xin Jiang, Zhi-Cheng Jing, Min Tang

**Affiliations:** ^1^State Key Lab of Cardiovascular Disease, Fu Wai Hospital, National Center for Cardiovascular Disease, Chinese Academy of Medical Sciences and Peking Union Medical College, Beijing, China; ^2^Department of Cardiology, Zhujiang Hospital, Laboratory of Heart Center, Southern Medical University, Guangzhou, China; ^3^State Key Laboratory of Complex Severe and Rare Diseases, Department of Cardiology, Peking Union Medical College Hospital, Chinese Academy of Medical Sciences and Peking Union Medical College, Beijing, China

**Keywords:** pulmonary hypertension, supraventricular tachycardia, radiofrequency catheter ablation, feasibility, outcome

## Abstract

**Background:**

Supraventricular tachycardia (SVT) occurs commonly and is strongly correlated with clinical deterioration in patients with pulmonary hypertension (PH). This study aimed to investigate the feasibility and long-term outcome of radiofrequency catheter ablation (RFCA) in PH patients with SVT.

**Materials and Methods:**

Consecutive PH patients with SVT who were scheduled to undergo electrophysiological study and RFCA between September 2010 and July 2019 were included. The acute results and long-term success of RFCA were assessed after the procedure.

**Results:**

In total, 71 PH patients with 76 episodes of SVT were analyzed. Cavotricuspid isthmus-dependent atrial flutter (*n* = 33, 43.5%) was the most common SVT type, followed by atrioventricular nodal reentrant tachycardia (*n* = 16, 21.1%). Of the 71 patients, 60 (84.5%) underwent successful electrophysiological study and were subsequently treated by RFCA. Among them, acute sinus rhythm was restored in 54 (90.0%) patients, and procedure-related complications were observed in 4 (6.7%) patients. Univariate logistic regression analysis showed that cavotricuspid isthmus-independent atrial flutter [odds ratio (OR) 25.00, 95% confidence interval (CI) 3.45–180.98, *p* = 0.001] and wider pulmonary artery diameter (OR 1.19, 95% CI 1.03–1.38; *p* = 0.016) were associated with RFCA failure. During a median follow-up of 36 (range, 3–108) months, 7 patients with atrial flutter experienced recurrence, yielding a 78.3% 3-year success rate for RFCA treatment.

**Conclusion:**

The findings suggest that RFCA of SVT in PH patients is feasible and has a good long-term success rate. Cavotricuspid isthmus-independent atrial flutter and a wider PAD could increase the risk for ablation failure.

## Introduction

Pulmonary hypertension (PH), a heterogeneous disease entity caused by various etiologies, is characterized by increased pulmonary vascular resistance and chronic right ventricular pressure overload (Simonneau et al., 2019). Despite the advancements of novel therapeutic agents, PH is still a progressive disease with poor prognosis and has a tendency to deteriorate over time (McLaughlin et al., 2015). While impairment of right ventricular-pulmonary artery coupling has been well recognized in PH, thus far, the role of the right atrium in maintaining normal cardiac output has been underestimated. The right atrium is able to assist with filling of the right ventricle at low pressure and is responsible for up to 30% of normal right ventricular output by contraction (Gaynor et al., 2005b). The structural and electrical remodeling of the right atrium in PH patients could contribute to the development of supraventricular tachycardia (SVT) and the immediate decrease of cardiac output (Gaynor et al., 2005a). Previous studies have demonstrated an approximate 20% incidence of SVT among PH patients, and SVT has been found to be independently associated with increased risks for clinical deterioration, hemodynamic instability, heart failure, and adverse prognosis (Tongers et al., 2007; Wen et al., 2014; Cannillo et al., 2015; Galiè et al., 2016; Middleton et al., 2019).

Regarding SVT management, rhythm control strategies have been more preferably recommended than simple rate control over the years, given that near normal physiological electrical activity is more beneficial for cardiac function, especially for PH patients (Page et al., 2016). Although antiarrhythmic medications are effective in rhythm control, they may also result in negative inotropic effects and important side effects (Doval et al., 1994; Amiodarone Trials Meta-Analysis Investigators, 1997). Depending on the precise mechanism involved in SVT revealed by electrophysiology (EP) study, radiofrequency catheter ablation (RFCA) can effectively permanently terminate SVT and free patients from antiarrhythmic medication use (Page et al., 2016). It is rational that a strategy based on RFCA to restore and maintain sinus rhythm could improve the symptoms and outcomes of PH patients, but potential obstacles from altered right atrial anatomy and electrophysiology often substantially prevent successful procedures (Tongers et al., 2007; Wen et al., 2014; Cannillo et al., 2015; Galiè et al., 2016). Therefore, this study aimed to investigate the feasibility and long-term success of RFCA in PH patients with SVT.

## Materials and Methods

### Study Population

Between September 2010 and July 2019, 96 suspected PH patients with SVT who were scheduled to undergo EP study were consecutively included for initial screening at Fuwai Hospital, a national center within the Chinese Academy of Medical Sciences. The episodes of SVT in the included patients were symptomatic with clinical worsening or right ventricular failure. In patients with accessible right heart catheterization (RHC) data, a mean pulmonary artery pressure ≥25 mmHg at rest was considered to be diagnostic for PH (Galiè et al., 2016). Otherwise, echocardiographic parameters were used to judge patients as having a high, intermediate or low probability of PH by two independent cardiologists specializing in PH (Bossone et al., 2013; Galiè et al., 2016). The PH patients were then classified into different subgroups according to the updated clinical classification algorithm (Simonneau et al., 2019). Episodes of SVT in PH patients were identified by electrocardiography, Holter monitoring, or EP study from previous medical records.

**FIGURE 1:**
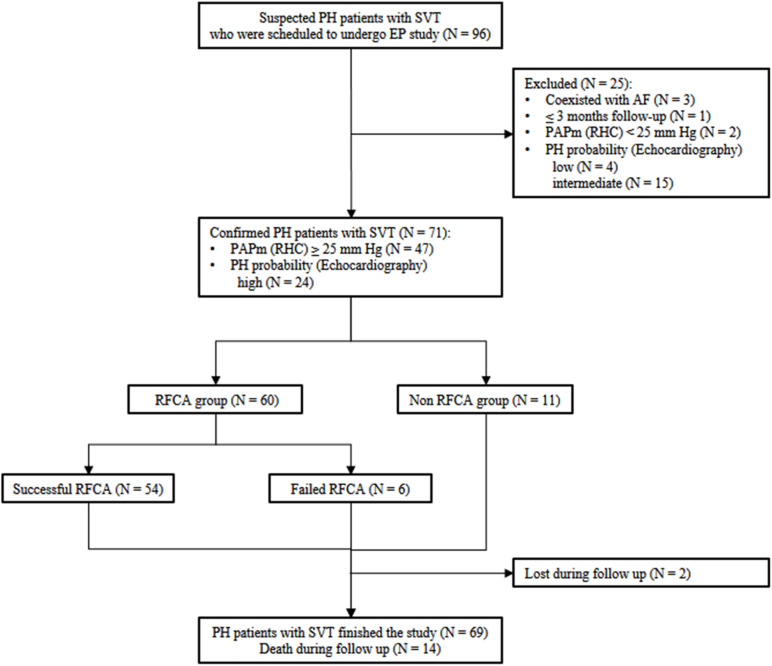
Flow chart for the inclusion-exclusion of the study population. PH, pulmonary hypertension; SVT, supraventricular tachycardia; EP, electrophysiology; RFCA, radiofrequency catheter ablation; RHC, right heart catheterization.

Of all suspected patients, 3 having atrial fibrillation and 1 with less than 3 months of follow-up were excluded. RHC was performed in 49 patients for hemodynamic assessment; of them, 47 fulfilled the definition of PH, while 2 did not. Among the 43 patients without RHC data, echocardiography was alternatively used to classify them as having a high (*n* = 24), intermediate (*n* = 15), or low (*n* = 4) probability of PH. As a result, a total of 71 PH patients (47 diagnosed by RHC and 24 by echocardiography) with SVT were ultimately included (Figure 1). The study conforms to the Declaration of Helsinki principles of medical ethics. The Ethics Committee of Fuwai Hospital approved this study, and all patients provided written informed consent.

### EP Study and Ablation Procedure

The EP study proceeded sequentially by measuring the conduction properties of the atrium, atrioventricular node, ventricle, and accessory pathways (if present); detecting the initiation of SVT; and determining the mechanism of tachycardia. Isoproterenol or atropine was used to facilitate induction if tachycardia could not be induced. According to the findings of the EP study, SVT was classified as atrioventricular nodal reentrant tachycardia (AVNRT), atrioventricular reentrant tachycardia (AVRT), focal atrial tachycardia, cavotricuspid isthmus (CTI)-dependent atrial flutter, or CTI-independent atrial flutter. If the EP study was not completed successfully, SVT was defined as SVT-unclassified. CTI dependence was assessed using entrainment mapping from the coronary sinus catheter and/or an ablation catheter advanced to the CTI. A pacing site was considered within the atrial flutter circuit if the postpacing interval minus the tachycardia cycle length was less than 30 ms when pacing 20–30 ms faster than the tachycardia cycle length. A three-dimensional electroanatomic mapping system (CARTO, Biosense Webster Inc., Diamond Bar, CA, United States or NavX Ensite Velocity, St. Jude Medical Inc., Milwaukee, WI, United States) was used in selected patients with AVNRT, AVRT or focal atrial tachycardia, depending on the operator’s choice. All atrial flutter cases used a three-dimensional electroanatomic mapping system. Entrainment mapping and three-dimensional activation sequence mapping were combined to differentiate atrial flutter. Catheter ablation was performed using a non-irrigated ablation catheter (Triguy, APT Medical Inc., Shenzhen, China or Safire, St. Jude Medical Inc.) in patients with AVNRT or AVRT. An irrigation catheter (ThermoCool Navi-Star/ThermoCool SmartTouch, Biosense Webster Inc., or FlexAbility/TactiCath, St. Jude Medical, Inc.) was used in focal atrial tachycardia or atrial flutter cases. In particular, irrigation catheter usage can lead to a risk for decompensated right heart failure in many PH patients with poor ventricular function; therefore, close hemodynamic monitoring during the process was imperative. If the procedure time was estimated to be prolonged, a Foley catheter was inserted to closely monitor intake and output. If the right atrium was enlarged, a long sheath was recommended to enhance catheter stability in AVNRT ablation. PH patients with cardiac transposition and CTI-dependent AFL required detailed mapping of the tricuspid annulus to ensure complete bidirectional block. The endpoints of catheter ablation were slow pathway modification in AVNRT, bidirectional absence of accessory pathway conduction in AVRT, and successful ablation of ectopic foci in focal atrial tachycardia bidirectional conduction block across the critical isthmus in atrial flutter.

The EP study and RFCA procedures performed in PH patients were somewhat different from those in the non-PH population. Some tips and tricks used in our center are summarized below. Before the procedure, careful preoperative evaluations, including echocardiography and cardiopulmonary function examination, helped determine the operation strategy. In particular, transesophageal echocardiography, which is poorly tolerated by PH patients, was safely avoided in some atrial flutter cases with a low risk of stroke and no imaging evidence of late-phase left atrial appendage filling defects (Zhai et al., 2018). Perioperative heart rate, blood pressure and pulse blood oxygen saturation were intensively monitored. Oxygen inhalation was usually needed. Generally, nasal catheters were used to supply oxygen. If the oxygen flow rate was over 6 L/min and the oxygen saturation was still low, a mask for oxygen inhalation was used to ensure that the oxygen saturation was not lower than 90%. All patients underwent vascular puncture in the groin area without ultrasound assistance, and lidocaine was used for local anesthesia. If the patient’s preoperative ECG suggested atrial flutter, intravenous intensive anesthesia was alternatively used considering the relatively large ablation range and long procedure time in atrial flutter cases. Anesthesia was administered by the EP members. One milligram of midazolam was injected intravenously, and fentanyl was continuously pumped intravenously (0.5 mg of fentanyl with 40 ml of saline, according to the patient’s weight, pump speed 10–18 ml/h). The purpose was to achieve mild to moderate sedation. At the same time, 5 mg of tropisetron was injected intravenously for antiemetic activity. Regarding optimal medication management, amiodarone was used properly to control the fast heart rate and concomitant unstable hemodynamics (i.e., systolic blood pressure lower than 90 mmHg). For severe PH cases, prostacyclin analogs were pumped continuously to control the high pulmonary artery pressure and prevent pulmonary hypertensive crisis. Diuretics were used sparingly to avoid the risk for electrolyte disturbances. During the procedures, heparin was used to keep the activated clotting time level between 250 and 300 s. Due to the frequent large right atrium and coronary sinus ostium in PH patients, coronary sinus electrode catheter placement using the femoral approach was favored. Coronary sinus catheter placement usually requires manually reshaping the catheter or using a long sheath. If the coronary sinus ostium was too large for the coronary sinus catheter to be stably sustained in atrial flutter cases, a coronary sinus catheter was placed in the right atrium appendage for mapping. Other multipolar electrode catheters were inserted percutaneously into the femoral vein and deployed into the patient’s bundle and right ventricular apex with the help of fluoroscopy.

### Data Collection and Follow-Up

Clinical data were obtained through the Fuwai Hospital Electronic Medical Record System. Baseline demographics, World Health Organization (WHO) functional class, 6 min walk distance, echocardiographic parameters, medications and laboratory data such as N-terminal pro brain natriuretic peptide (NT-proBNP) were collected at admission. Acute outcomes of RFCA included acute sinus rhythm restoration and procedure-related complications were recorded. Complications defined by the operation itself included atrioventricular block, sick sinus syndrome, cardiac perforation or tamponade, stroke or transient ischemic attack, and vascular access issues such as hematoma, pseudoaneurysm, or arteriovenous fistula.

The long-term success of RFCA was defined in the absence of SVT recurrence during follow-up. The follow-up data were collected until the last visit. Follow-up visits were regularly performed at the outpatient clinic or hospital or by telephone to obtain information on vital status, SVT recurrence, medication, etc. The first follow-up was performed 3 months after the first RFCA and then every 3–6 months thereafter. Patients were considered lost to follow-up if they did not answer their telephone after 3 separate attempts.

### Statistical Analysis

Continuous data are presented as the mean with standard deviation (SD) or median with interquartile range (IQR, 25–75th percentiles), as appropriate. Dichotomous data are expressed as numbers with percentages. The 6 min walking distances of 5 patients were missing and were therefore imputed with the mean value. Comparison of continuous variables between 3 different RFCA groups was performed with one-way analysis of variance (ANOVA). Comparison of continuous variables between the successful RFCA and failed RFCA groups was conducted with Student’s *t*-test. Dichotomous data were compared using the chi-squared test or Fisher’s exact test as appropriate. Univariate binary logistic regression analysis was utilized to evaluate the relationship between baseline characteristics and RFCA failure. In consideration of the small sample size, multivariate analysis was not performed to avoid overfitting. A two-tailed *p* < 0.05 was considered significant. All statistical analyses were performed using R version 3.6.1 (R Foundation for Statistical Computing, Vienna, Austria).

## Results

### Baseline Characteristics

The mean age of the 71 patients was 43.9 ± 14.9 years, and there was a slight predominance of females (56.3%) (Table 1). The most common PH types were pulmonary arterial hypertension associated with congenital heart disease (*n* = 38, 53.5%) and idiopathic and heritable pulmonary arterial hypertension (*n* = 22, 31%) (Table 2). The patients were more often WHO class III/IV (*n* = 43, 60.6%) and exhibited elevated systolic pulmonary artery pressure (80.4 ± 25.7 mmHg), as measured by echocardiography. Among patients who received RHC examination, the invasive systolic pulmonary artery pressure and mean pulmonary artery pressure were 84.3 ± 25.9 and 55.7 ± 16.9 mmHg, respectively. Antiarrhythmic drugs were administered, including amiodarone/sotalol (*n* = 28, 39.4%), β blockers (*n* = 14 19.7%) and propafenone (*n* = 4, 5.6%). PH-targeted therapies were commonly prescribed (*n* = 52, 73.2%) and included phosphodiesterase type 5 inhibitors (*n* = 43, 60.6%), endothelin receptor antagonists (*n* = 36, 50.7%), and prostacyclin analogs/receptor agonists (*n* = 10, 14.1%).

**TABLE 1:** Baseline characteristics of the study population in relation to different outcomes of RFCA.

	All (*n* = 71)	Successful RFCA (*n* = 54)	Failed RFCA (*n* = 6)	None RFCA (*n* = 11)	*P*-value^‡^	*P*-value^§^
Age (years)	43.9 ± 14.9	43.5 ± 14.6	41.8 ± 17.0	46.8 ± 16.4	0.757	0.790
Female	40 (56.3)	29 (53.7)	4 (66.7)	7 (63.6)	0.719	0.545
BMI (kg/m^2^)	22.0 ± 3.9	22.2 ± 4.2	21.4 ± 32.7	21.2 ± 3.1	0.718	0.624
Diabetes	2 (2.8)	2 (2.8)	0 (0)	0 (0)	0.573	0.632
Hypertension	7 (9.9)	7 (13.0)	0 (0)	0 (0)	0.131	0.348
Dyslipidemia	4 (5.6)	3 (4.2)	0 (0)	1 (9.1)	0.635	0.554
WHO functional class III-IV	43 (60.6)	32 (56.6)	4 (66.7)	7 (63.6)	0.842	0.759
PAPs^∗^ (mmHg)	80.4 ± 25.7	78.0 ± 25.2	82.2 ± 26.3	92.1 ± 27.4	0.282	0.704
PAPs^†^ (mmHg)	84.3 ± 25.9	80.5 ± 26.5	93.3 ± 20.6	96.3 ± 22.7	0.219	0.420
PAPm^†^ (mmHg)	55.7 ± 16.9	53.5 ± 17.1	65.7 ± 12.0	61.2 ± 16.5	0.277	0.237
6MWD (m)	340 ± 94	354 ± 89	314 ± 120	279 ± 88	0.120	0.370
NT-proBNP (pg/ml)	1,925 (880, 2,634)	1,996 (887, 2,793)	1,736 (932, 2,500)	1,706 (796, 2,245)	0.708	0.979
Total bilirubin (U/L)	30.9 ± 24.1	29.7 ± 21.8	39.1 ± 25.7	31.7 ± 33.8	0.668	0.332
Uric acid (μmol/L)	415.0 ± 155.2	419.1 ± 152.4	466.7 ± 142.5	367.8 ± 175.8	0.430	0.470
LVEDD (mm)	41.1 ± 11.4	40.2 ± 9.0	46.0 ± 15.9	42.7 ± 17.7	0.445	0.185
RVD (mm)	38.3 ± 11.0	39.0 ± 10.6	41.7 ± 13.2	33.1 ± 38.3	0.202	0.542
LVEF (%)	60.8 ± 9.6	61.6 ± 9.4	58.2 ± 7.5	58.9 ± 11.6	0.552	0.403
PAD (mm)	32.5 ± 6.1	31.9 ± 5.2	38.7 ± 8.9	32.5 ± 7.4	0.032	0.006
RA dilatation	58 (81.7)	46 (85.2)	4 (66.7)	8 (72.7)	0.413	0.248
PH-targeted therapy	52 (73.2)	41 (75.9)	4 (66.7)	7 (63.6)	0.654	0.619
ERA	36 (50.7)	27 (50.0)	4 (66.7)	5 (45.5)	0.685	0.438
PDE5-i	43 (60.6)	35 (64.8)	2 (33.3)	6 (54.5)	0.303	0.132
Prostacyclin analogs/receptor agonists	10 (14.1)	7 (13.0)	2 (33.3)	1 (9.1)	0.421	0.185
β blocker	14 (19.7)	8 (14.8)	2 (33.3)	4 (36.4)	0.208	0.248
Amiodarone/sotalol	28 (39.4)	21 (38.9)	3 (50)	4 (36.4)	0.850	0.598
Propafenone	4 (5.6)	2 (3.7)	2 (33.3)	0 (0)	0.051	0.046

Data are presented as n (%), mean (SD), or median (IQR). *Measured by echocardiography. ^†^Measured by right heart catheterization. ^‡^p-value for overall comparisons. ^§^p-value for successful RFCA vs. failed RFCA. RFCA, radiofrequency catheter ablation; BMI, body mass index; WHO, World Health Organization; PAPs, systolic pulmonary artery pressure; PAPm, mean pulmonary artery pressure; 6MWD, 6 min walk distance; NT-proBNP, N-terminal pro B type natriuretic peptide; LVEDD, left ventricular and diastolic diameter; RVD, right ventricular diameter; LVEF, left ventricular ejection fraction; PAD, pulmonary artery diameter; RA, right atrium; PH, pulmonary hypertension; ERA, endothelin receptor antagonists; PDE5-I, phosphodiesterase type 5 inhibitors.

**TABLE 2:** Classification of pulmonary hypertension among the study population.

	All (*n* = 71)	Successful RFCA (*n* = 54)	Failed RFCA (*n* = 6)	None RFCA (*n* = 11)
Idiopathic and heritable pulmonary arterial hypertension	22 (31.0%)	17 (31.5%)	1 (16.7%)	4 (18.2%)
Pulmonary arterial hypertension associated with congenital heart disease	38 (53.5%)	28 (51.9%)	5 (83.3%)	5 (45.5%)
Pulmonary hypertension due to left heart disease	4 (5.6%)	2 (3.7%)	0 (0.0%)	2 (50.0%)
Pulmonary hypertension due to lung disease/hypoxia	1 (1.4%)	0 (0%)	0 (0%)	0 (0%)
Chronic thromboembolic pulmonary hypertension	4 (5.6%)	4 (7.4%)	0 (0%)	0 (0%)
Pulmonary hypertension due to pulmonary vasculitis	2 (2.8%)	2 (2.7%)	0 (0%)	0 (0%)

*Data are presented as the n (%). RFCA, radiofrequency catheter ablation.*

### Types of SVT Determined by EP Study

**FIGURE 2:**
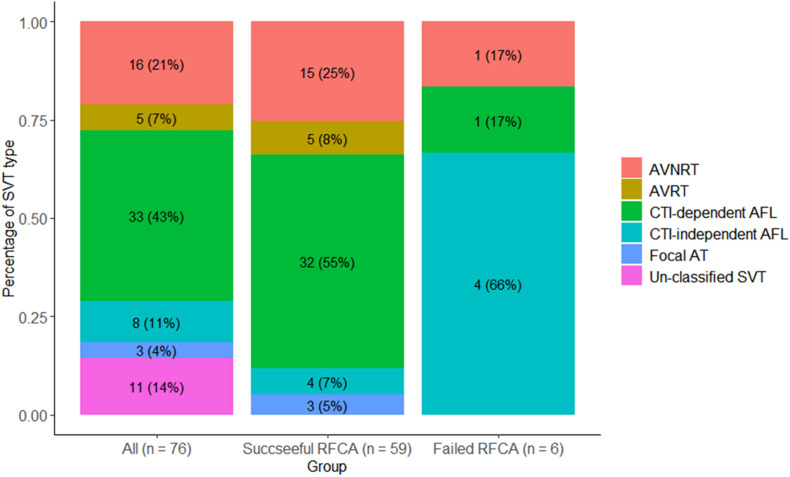
Types of SVT. AVNRT, atrioventricular nodal reentrant tachycardia; AVRT, atrioventricular reentrant tachycardia; CTI, cavotricuspid isthmus; AFL, atrial flutter; AT, atrial tachycardia; SVT, supraventricular tachycardia.

Among the 71 patients, 76 episodes of tachycardia were recorded. Of them, 65 tachycardias were confirmed by EP study, and 11 tachycardias were identified by clinically documented electrocardiography due to unsuccessful CS catheter placement caused by a large right atrium and abnormal coronary sinus anatomy (*n* = 1), non-inducible SVT (*n* = 5), or non-sustainable SVT that precluded adequate mapping and ablation (*n* = 5). The detailed types of SVT are summarized in Figure 2. Among all tachycardias, CTI-dependent atrial flutter was the most common type (*n* = 33; 43.5%), followed by AVNRT (*n* = 16; 21.1%).

### Acute Outcomes of RFCA

A total of 60 patients underwent RFCA treatment, of which 54 patients received successful ablation while 6 did not, yielding a rate of acute sinus rhythm restoration of 90%. All 71 patients were accordingly classified into a successful RFCA group (*n* = 54), a failed RFCA group (*n* = 6), and a non-RFCA group (*n* = 11). The top 2 types of SVT in the successful RFCA group were still CTI-dependent atrial flutter (*n* = 32; 54.2%) and AVNRT (*n* = 15; 25.4%). In contrast, the SVT types of the failed RFCA group were CTI-independent atrial flutter (*n* = 4), CTI-dependent atrial flutter (*n* = 1) and AVNRT (*n* = 1) (Figure 2). CTI-independent atrial flutter was far more common in the failed group than in the successful group (66.6 vs. 6.8%; *p* < 0.01). Additionally, the pulmonary artery diameter in the failed group was wider than that in the successful group (38.7 ± 8.9 vs. 31.9 ± 5.2 mm, *p* = 0.006) (Table 1). According to univariate logistic regression analysis (Table 3), CTI-independent atrial flutter [odds ratio (OR) 25.00, 95% confidence interval (CI) 3.45–180.98, *p* = 0.001] and pulmonary artery diameter (OR 1.19, 95% CI 1.03–1.38, *p* = 0.016) were associated with RFCA failure.

**TABLE 3:** Univariate logistic analysis of predictors of failed RFCA.

Variables	OR (95% CI)	*P*-value
Age, years	0.99 (0.94–1.05)	0.786
Female vs. male	1.72 (0.29–10.22)	0.549
BMI, kg/m^2^	0.95 (0.75–1.21)	0.670
CTI independent-AFL, yes vs. no	25.00 (3.45, 180.98)	0.001
PAPs, mmHg	1.01 (0.98–1.04)	0.606
PAPm, mmHg	1.01 (0.96–1.07)	0.728
WHO class, III-IV vs. I-II	1.15 (0.44–3.02)	0.782
RVD, mm	1.02 (0.95–1.11)	0.569
LVEDD, mm	1.05 (0.98–1.13)	0.191
PAD, mm	1.19 (1.03–1.38)	0.016
6MWD, m	1.00 (0.98–1.01)	0.367
NT-proBNP, pg/ml	1.00 (0.99–1.00)	0.690
Total bilirubin, U/L	1.02 (0.98–1.05)	0.334
Creatinine, μmol/L	0.95 (0.88–1.02)	0.136

*CTI independent-AFL, cavotricuspid isthmus-independent atrial flutter; OR, odds ratio; CI, confidence interval; other abbreviations as in Table 1.*

The perceived reasons for ablation failure are as follows. In a patient with AVNRT, there was substantial risk for atrioventricular block during ablation due to a huge right atrium and coronary sinus ostium, so the operation was abandoned for safety reasons. In a patient with CTI-dependent atrial flutter, it was difficult to block the isthmus even after repeat ablation because of the large right atrium and long CTI. Considering that the treatment was poorly tolerated by the patient, the operation was appropriately suspended. In another four patients with CTI-independent atrial flutter, the operators were unable to determine the reentrant circuit by repeated mapping due to unstable tachycardia cycle length. After repeated failed attempts of ablation to stop the tachycardia, especially considering that one of the patients even suffered stroke, the operations were terminated.

Four patients from the RFCA group experienced the following complications: third-degree atrioventricular block (*n* = 1), stroke (*n* = 1), arteriovenous fistula (*n* = 1), and pseudoaneurysm of the femoral artery (*n* = 1) (Table 4). In summary, the complication rate in the RFCA group was 6.7%. In one PH patient with AVNRT having a third-degree atrioventricular block after RFCA, it was speculated that the large right atrium and very high systolic pulmonary artery pressure (120 mmHg) might have affected catheter stability so that ablating the antegrade atrioventricular node slow pathway was complicated by catheter migration and resultant ablation of all antegrade conduction. One week later, a third-degree atrioventricular block persisted in this patient, and a pacemaker was implanted. The patient with procedure-related stroke was found to have atrial septal defects with right to left shunting, and the induced SVT was CTI-independent atrial flutter that localized to the right atrium. The procedure time was prolonged while heparin was administered according to a standard protocol. It was hypothesized that atrial thromboembolism led to the occurrence of stroke after a CT scan excluded the possibility of hemorrhage. Fortunately, this patient had good functional recovery with minimal residual deficit of the left lower extremity. The patient with a 2 mm diameter arteriovenous fistula was treated using normal compression, and the arteriovenous fistula occluded spontaneously during follow-up. In the patient with pseudoaneurysm, conservative treatment was ineffective, and percutaneous balloon occlusion was then used to achieve successful closure of the pseudoaneurysm. Using ultrasound to guide vascular puncture may help reduce the risk of vascular complications.

**TABLE 4:** Complications associated with the ablation procedure.

	All (*n* = 58)	Successful RFCA (*n* = 52)	Failed RFCA (*n* = 6)
Total	4 (6.7)	3 (5.6)	1 (16.7)
Arterio-venous fistula	1 (1.7)	1 (1.9)	0 (0)
Pseudoaneurysm	1 (1.7)	1 (1.9)	0 (0)
Atrioventricular block	1 (1.7)	1 (1.9)	0 (0)
Stroke	1 (1.7)	0 (0)	1 (16.7)

*Data are presented as the n (%).*

### Long-Term Outcome of RFCA

During a median of 36 (range, 3–108) months of follow-up, 6 patients with CTI-dependent atrial flutter and 1 patient with CTI-independent atrial flutter experienced recurrent tachycardias, and the electrocardiographic findings of each patient were identical to the documented preablation tachycardia. Thus, the long-term success rate of RFCA was 78.3%. Among these 7 patients, two with CTI-dependent atrial flutter and 1 with CTI-independent atrial flutter underwent a second successful ablation. Of the 3 patients who had a second ablation, one patient with CTI-dependent atrial flutter required a third ablation that was successful. After repeated RFCA procedures, 50 (83.3%) patients achieved successful RFCA (Supplementary Figure 1).

### Sensitivity Analysis

Considering that patients with pulmonary hypertension due to left heart disease are more commonly treated with catheter ablation for atrial arrhythmias, we conducted a sensitivity analysis after excluding patients with pulmonary hypertension due to left heart disease (*n* = 4). Among the remaining patients, 58 patients underwent RFCA treatment, of which 52 patients received successful ablation while 6 did not, yielding a rate of acute sinus rhythm restoration of 89.7% (52/58). According to univariate logistic regression analysis (Supplementary Table 1), CTI-independent atrial flutter and pulmonary artery diameter were still associated with RFCA failure. Four patients experienced complications, resulting in a 6.9% (54/58) complication rate (Supplementary Table 2). During follow-up, the long-term success rate of RFCA was 77.6% (45/58). These results were comparable with the results of the main analyses.

## Discussion

The major findings of the study were as follows: (1) CTI-dependent atrial flutter and AVNRT were the 2 most common types of SVT in this cohort; (2) RFCA was feasible in PH patients for the treatment of SVT, and the long-term RFCA results remained favorable; and (3) CTI-independent atrial flutter and a wider pulmonary artery diameter could increase the risk of ablation failure.

As reported, atrial fibrillation and atrial flutter accounted for most incident SVTs in PH patients (Wen et al., 2014; Tongers et al., 2007). In the current study, CTI-dependent atrial flutter and AVNRT were the most common SVT types because we excluded patients with atrial fibrillation. Atrial fibrillation ablation was rarely performed in PH patients because of the underlying risks. On the one hand, transseptal puncture might lead to iatrogenic atrial septal defects, particularly with the use of the cryoballoon ablation system, which requires a larger sheath for delivery. A significant residual atrial septal defect in PH patients with elevated right heart filling pressures may result in clinically significant intracardiac right to left shunting and hypoxemia and an increased risk of paradoxical embolism (Wanamaker et al., 2018). On the other hand, atrial fibrillation ablation with bilateral circumferential pulmonary vein isolation may cause pulmonary vein edema or even pulmonary vein stenosis. These conditions could increase the pulmonary venous and arterial pressures and predispose patients to clinical deterioration.

A number of potential factors contributing to SVT susceptibility have been discussed in the past decade, including autonomic nervous system disturbance, electrical remodeling and myocardial ischemia (Medi et al., 2012; Cirulis et al., 2019). In particular, chronic pressure overload from PH could lead to remodeling of the right heart with hypertrophy and fibrosis, the latter of which provided the trigger substrate for CTI-independent atrial flutter (Medi et al., 2012). Furthermore, the hypertensive pulmonary vasculature eventually induced enlargement of the right atrium and coronary sinus ostium, which has been shown to be associated with the onset of AVNRT and CTI-dependent atrial flutter (Doig et al., 1995; Okumura et al., 2004; Ezhumalai et al., 2014). More interestingly, the two abovementioned arrhythmias might be involved in a common area of perinodal atrium adjacent to coronary sinus ostium in their tachycardia circuits (Okumura et al., 2004).

A few studies have investigated the efficacy of RFCA for SVT in PH patients. In these studies, the vast majority of SVTs were usually CTI-dependent atrial flutter, and their results demonstrated that CTI ablation in PH patients was safe and effective (Berdjis et al., 1996; Showkathali et al., 2011; Bradfield et al., 2012; Luesebrink et al., 2012). Moreover, these studies only reported acute clinical improvement after RFCA treatment but no long-term outcome. In the current study enrolling a larger population of PH patients with SVT, the cohort included not only CTI-dependent atrial flutter but also CTI-independent atrial flutter, focal atrial tachycardia, AVNRT, and AVRT. The results demonstrated that in PH patients with SVT, RFCA was feasible with an acute success rate of 90%, which was in line with the results from the non-PH population (Keegan et al., 2015). During follow-up, 7 patients with atrial flutter experienced recurrence of the same preablation tachycardias. The long-term ablation success rates were 78.3% for all types of SVT and 70.7% for atrial flutter, and the data were slightly lower than the 80% rate of sinus rhythm maintenance reported in the non-PH population (Natale et al., 2000). In summary, these findings favor ablation treatment as first-line therapy in the management of SVT in PH patients.

Although ablation treatment in PH patients was favorable, there were still some significant differences in procedures compared with those in non-PH patients. A higher prevalence of CTI-independent atrial flutter and wider pulmonary artery diameter have been observed in PH patients with failed RFCA. The mechanism of CTI-independent atrial flutter is complex, especially in PH patients, due to extensive atrial remodeling and fibrosis (Medi et al., 2012). Therefore, RFCA of CTI-independent atrial flutter in PH patients is still challenging and prone to failure. The wider pulmonary artery diameter reflected high pulmonary artery pressure and long-standing PH, which were associated with reduced tissue voltage and regions of electrical silence (Medi et al., 2012). The enlargement of the right atrium and abnormal coronary sinus ostium collectively increased the difficulty of catheter cannulation and ablation and increased the risk for procedure-related complications. To reduce the risk of complications, it is particularly important to enhance catheter stability using a long sheath as appropriate and keep the activated clotting time in an adequate range when the operation time is beyond that expected. Some tips and tricks listed in the methods deserve serious attention to reduce the risk of complications.

The current study has several limitations. First, this study was retrospective and single-centered. Postablation changes in right heart catheterization parameters, echocardiography results and other functional measurements were incomplete, which made it difficult to assess the immediate effect of RFCA on clinical outcome. However, this study still provided important information about the long-term outcome of RFCA in PH patients with SVT. Second, as a real-world study, there was a certain degree of heterogeneity among our patients with pulmonary hypertension. We performed the corresponding sensitivity analysis, and the result was stable. Considering the relatively small sample size of our study, a study focusing on a more discrete population with a larger sample size is needed in the future. Third, the lack of uniform protocols due to the observational nature of our study might influence the ablation results. However, the ablation strategy for each patient was routinely discussed among the members of an experienced electrophysiologist team in our center, which could decrease the heterogeneity of operating skills. Meanwhile, three-dimensional electroanatomic mapping was only used in certain cases, which may partly influence the study results. Fourth, although the sample size in this study was the largest in this field thus far, it was still too small to address the question of safety. Therefore, we only reported the complication rate in our study, which was relatively low. We also only performed univariate analysis considering the limited number of events in our study. These findings should be considered hypothesis-generating rather than robust evidence. A prospective study with a larger sample size is warranted to further explore the role of RFCA in SVT in PH patients.

In conclusion, RFCA of SVT in PH patients is feasible and has a good long-term success rate. Cavotricuspid isthmus-independent atrial flutter and wider PAD could increase the risk for ablation failure. A prospective study with a larger sample size is needed to further verify these findings.

## Data Availability Statement

The raw data supporting the conclusions of this article will be made available by the authors, without undue reservation.

## Ethics Statement

The studies involving human participants were reviewed and approved by the Ethical Committee of Fuwai Hospital. The patients/participants provided their written informed consent to participate in this study.

## Author Contributions

Z-CJ and MT conceived and designed research. BZ, Z-QZ, S-XW, Y-ZM, F-YY, Y-JQ, Y-ZJ, and MT accomplished the EP or RFCA procedure. BZ and Y-JZ collected and analyzed the data and wrote the manuscript. XG, X-QX, XJ, Z-CJ, and MT revised the manuscript. All authors read and approved the final manuscript.

## Conflict of Interest

The authors declare that the research was conducted in the absence of any commercial or financial relationships that could be construed as a potential conflict of interest.
