# Supplementary material for: Radiofrequency Catheter Ablation of Supraventricular Tachycardia in Patients With Pulmonary Hypertension: Feasibility and Long-Term Outcome
Source: Front Physiol. 2021 Jun 18;12:674909. doi: 10.3389/fphys.2021.674909 (PMC8249814; doi:10.3389/fphys.2021.674909)
Supplement: Supplementary file 1 [file Data_Sheet_1.docx]

**Supplementary Materials**

**Radiofrequency Catheter Ablation of** **Supraventricular Tachycardia in Patients with Pulmonary Hypertension: Feasibility and Long-Term Outcome**

*Bin Zhou, Yong-Jian Zhu, Zheng-Qin Zhai, Si-Xian Weng, Ya-Zhe Ma, Feng-Yuan Yu, Ying-Jie Qi, Yi-Zhou Jiang, Xin Gao^1^ Xi-Qi Xu, Xin Jiang, Zhi-Cheng Jing and Min Tang*

**Table S1 Univariate logistic analysis of predictors of failed RFCA excluding patients with pulmonary hypertension due to left heart disease**

| **Varibles** | **OR (95% CI)** | **p Value** |
| --- | --- | --- |
| Age, years | 1.00 (0.94-1.06) | 0.874 |
| Female vs. male | 1.71 (0.29-10.22) | 0.554 |
| BMI, kg/m^2^ | 0.95 (0.75-1.20) | 0.642 |
| CTI independent-AFL, yes vs. no | 25.00 (3.45, 180.88) | 0.001 |
| PAPs, mmHg | 1.01 (0.97-1.04) | 0.683 |
| PAPm, mmHg | 1.01 (0.95-1.06) | 0.815 |
| WHO class, III-IV vs. I-II | 1.15 (0.44-3.03) | 0.812 |
| RVD, mm | 1.02 (0.94-1.11) | 0.612 |
| LVEDD, mm | 1.05 (0.98-1.13) | 0.185 |
| PAD, mm | 1.19 (1.03-1.37) | 0.018 |
| 6MWD, m | 1.00 (0.98-1.01) | 0.362 |
| NT-proBNP, pg/ml | 1.00 (1.00-1.00) | 0.650 |
| Total bilirubin, U/L | 1.02 (0.98-1.05) | 0.363 |
| Creatinine, μmol/L | 0.95 (0.89-1.02) | 0.148 |

**Table S2 Complications associated with ablation procedure excluding patients with pulmonary hypertension due to left heart disease**

|  | **ALL**  **(n = 58)** | **Successful RFCA**  **(n = 52)** | **Failed RFCA**  **(n = 6)** |
| --- | --- | --- | --- |
| Total | 4 (6.9) | 3 (5.8) | 1 (16.7) |
| Arterio-venous fistula | 1 (1.7) | 1 (1.9) | 0 (0) |
| Pseudoaneurysm | 1 (1.7) | 1 (1.9) | 0 (0) |
| Atrioventricular block | 1 (1.7) | 1 (1.9) | 0 (0) |
| Stroke | 1 (1.7) | 0 (0) | 1 (16.7) |

Legend: Data are presented as the n (%).


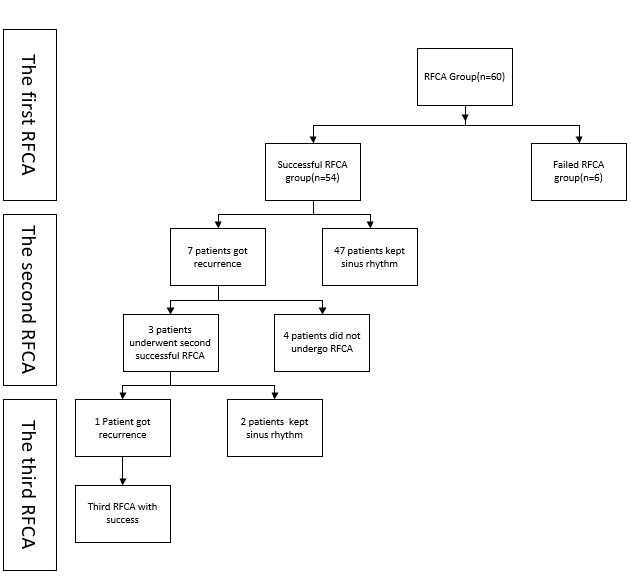


**FIGURE S1 |** The long-term efficiency of radiofrequency catheter ablation after repeated procedures.
